# Dissemination of imipenem-resistant *Acinetobacter baumannii* with new plasmid-borne *bla*_OXA-72_ in Taiwan

**DOI:** 10.1186/1471-2334-13-319

**Published:** 2013-07-13

**Authors:** Shu-Chen Kuo, Su-Pen Yang, Yi-Tzu Lee, Han-Chuan Chuang, Chien-Pei Chen, Chi-Ling Chang, Te-Li Chen, Po-Liang Lu, Po-Ren Hsueh, Chang-Phone Fung

**Affiliations:** 1 Institute of Clinical Medicine, National Yang-Ming University, School of Medicine, No.155, Sec. 2, Linong Street, Beitou District, Taipei 112, Taiwan; 2 National Institute of Infectious Diseases and Vaccinology, National Health Research Institutes, No. 35, Keyan Rd., Zhunan, Miaoli County 350, Taiwan; 3 Division of Infectious Diseases, Department of Medicine, Taipei Veterans General Hospital, No. 201, Sec. 2, Shipai Rd., Beitou District, Taipei 112, Taiwan; 4 Institute of Emergency and Critical Care Medicine, National Yang-Ming University, No.155, Sec. 2, Linong Street., Beitou District, Taipei 112, Taiwan; 5 Emergency Department, Taipei Veterans General Hospital, No. 201, Sec. 2, Shipai Rd., Beitou District, Taipei 112, Taiwan; 6 Division of Infectious Disease, Department of Medicine, Buddhist Taipei Tzu Chi General Hospital, No. 289, Jianguo Rd., Xindian Dist., New Taipei City 231, Taiwan; 7 Department of Internal Medicine, Kaohsiung Medical University Hospital, Kaohsiung, No. 100, Zihyou 1st Rd., Kaohsiung City 807, Taiwan; 8 Department of Laboratory Medicine, National Taiwan University Hospital, National Taiwan University College of Medicine, No. 1, Changde St., Zhongzheng District, Taipei City 100, Taiwan

**Keywords:** Imipenem-resistant, *Acinetobacter baumannii*, Carbapenemase, *Bla*_OXA-72_

## Abstract

**Background:**

The systemic surveillance of imipenem-resistant *Acinetobacter baumannii* (IRAB) from multicenters in Taiwan revealed the emergence of isolates with *bla*_OXA-72_. This study described their genetic makeup, mechanism of spread, and contribution to carbapenem resistance.

**Methods:**

Two hundred and ninety-one non-repetitive isolates of *A. baumannii* were collected from 10 teaching hospitals from different geographical regions in Taiwan from June 2007 to September 2007. Minimal inhibitory concentrations (MICs) were determined by agar dilution. Clonality was determined by pulsed-field gel electrophoresis. Plasmid was extracted and digested by restriction enzymes, and subsequently analyzed by electrophoresis and Southern blot for *bla*_OXA-72_. The flanking regions of *bla*_OXA-72_ were determined by inverse PCR. The contribution of *bla*_OXA-72_ to imipenem MIC was determined by transforming plasmids carrying *bla*_OXA-72_ into imipenem-susceptible *A. baumannii.*

**Results:**

Among 142 IRAB in Taiwan, 27 harbored *bla*_OXA-72_; 22 originated from Southern Taiwan, 5 from Central Taiwan, and none from Northern Taiwan. There were two major clones. The *bla*_OXA-72_ was identified in the plasmids of all isolates. Two genetic structures flanking plasmid-borne *bla*_OXA-72_ were identified and shared identical sequences in certain regions; the one described in previous literature was present in only one isolate, and the new one was present in the remaining isolates. Introduction of *bla*_OXA-72_ resulted in an increase of imipenem MIC in the transformants. The overexpression of *bla*_OXA-72_ mRNA in response to imipenem further supported the contribution of *bla*_OXA-72._

**Conclusions:**

In conclusion, isolates with new plasmid-borne *bla*_OXA-72_ were found to be disseminated successfully in Southern Taiwan. The spread of the resistance gene depended on clonal spread and dissemination of a new plasmid. *Bla*_OXA-72_ in these isolates directly led to their imipenem-resistance.

## Background

*Acinetobacter baumannii* has become an important nosocomial pathogen throughout the world because of its drug resistant phenotype [1]. The resistance to carbapenems is the most troublesome due to a limited number of efficacious drugs and the association with poor prognosis [2]. The mortality associated with imipenem-resistant *A. baumannii* (IRAB) is higher than that caused by susceptible strains, which is attributable to the higher rate of inappropriate antimicrobial therapy [3,4]. Although modification of antibiotic targets, overexpression of efflux pumps, and loss of porins leads to carbapenem resistance, the main mechanism in the *Acinetobacter* species is production of carbapenemases, including Ambler class B metallo-β-lactamases and class D β-lactamases [5]. Compared with other *Acinetobacter* species, class D β-lactamases in *A. baumannii,* including the intrinsic *bla*_OXA-51-like_ as well as acquired *bla*_OXA-23-like_, *bla*_OXA-24-like_, *bla*_OXA-58-like_, and *bla*_OXA-143_ genes are more commonly identified as the cause of resistance [5,6]. The distribution of class D β-lactamases varies among countries, even among regions in the same country [7]. Isolates with *bla*_OXA-23_ spread over a wide geographical range [8], whereas those carrying *bla*_OXA-24_, though relatively less common, have emerged in France, Italy, South Korea, and Brazil [9-12]. In Taiwan, one pilot study described the spread of isolates with *bla*_OXA-72_, a member of the *bla*_OXA-24_ family, within a single medical center [13]. Thus, in this study, we collected clinical isolates of *A. baumannii* from 10 medical centers in different areas in Taiwan during 2007 and found the different geographic distribution of isolates carrying *bla*_OXA-24/72_. Therefore, this multicenter study aimed to describe their genetic makeup, mechanism of spread, and contribution to carbapenem resistance in *A. baumannii* in Taiwan.

## Methods

### Bacterial identification and antimicrobial susceptibility testing

Two hundred and ninety-one non-repetitive isolates of *A. baumannii* were retrospectively collected from 10 teaching hospitals from different geographical regions in Taiwan from June 2007 to September 2007. Four hospitals are located in Northern (N1-N4) Taiwan, 3 are located in Central (C1-C3) Taiwan, and 3 are located in Southern (S1-S3) Taiwan (Additional File 1). All isolates were taken as part of standard patient care and investigators retrospectively collected these isolates. This microbiological study without patient data was exempt from full review by our institutional review boards.

All isolates were identified to the species level using a multiplex PCR method to confirm the specific intergenic spacer region in *A. baumannii*[14]. The minimal inhibitory concentration (MIC) of imipenem, meropenem, ticarcillin, piperacillin, ceftazidime, cefepime, sulbactam, ciprofloxacin, and colistin was determined using the agar dilution method according to the guidelines provided by the Clinical and Laboratory Standards Institute (CLSI) [15]. The testing and interpretation were in accordance with the guidelines of CLSI breakpoints or manufacturer’s instructions. *A. baumannii* isolates that were resistant to imipenem were subjected to the experiments described below.

### Identifying Ambler class B metallo-β-lactamases and class D β-lactamases

PCR methods were used to detect *bla*_OXA-23-like_, *bla*_OXA-24-like_, *bla*_OXA-58-like_, *bla*_IS*Aba1*-OXA51-like_, *bla*_OXA-143,_*bla*_IMP-like_, *bla*_VIM-like_, *bla*_GIM-1_, *bla*_SPM-1_ and *bla*_SIM-1_ as previously described [6,16,17]. The PCR products were then sequenced at Mission Biotech, Taipei, Taiwan. Primers of IS*Aba1*F and IS*Aba1*R were used to detect the presence of IS*Aba1,* whereas the IS*Aba1*F and OXA-likeR primers against the different genes described above were used to confirm the presence of IS*Aba1* upstream of the carbapenemase gene [16].

### Determination of clonality by pulsed-field gel electrophoresis of isolates with *bla*_OXA-72_

The clonality of strains with *bla*_OXA-72_ was determined by pulsed-field gel electrophoresis after digestion with *Apa*I as previously described [18]. The stained gel was photographed and analyzed by BioNumerics software (Applied Maths) to generate a dendrogram of relatedness among these isolates. Isolates with similarity > 85% was designated as one clone.

### Determination of the plasmid localization of *bla*_OXA-72_ by Southern blot

The plasmid localization of *bla*_OXA-72_ was visualized as previous reported [19]. The plasmid was extracted with a plasmid DNA Miniprep Kit (Bioman, Taipei, Taiwan) or a plasmid Maxiprep Kit (Qiagen, Valencia, CA). The extracts were treated with *Eco*RI and subjected to electrophoresis. After transfer to the hybridization transfer membrane (PerkinElmer, Boston, MA) and hybridization with a PCR-generated probe derived from primers targeting *bla*_OXA-72_ (Additional File 2), the band was visualized with a digoxigenin (DIG) DNA labeling and detection kit (Roche Diagnostics, Basel, Switzerland) according to the manufacturer’s instructions.

### Sequencing of flanking region of plasmid-borne *bla*_OXA-72_ by inverse PCR

Inverse PCR was performed as previously reported [20]. Briefly, the plasmid extracted from isolates carrying *bla*_OXA-72_ was digested with *Eco*RI, *Hin*dIII, or *Bg*lII, respectively. After overnight incubation and subsequent purification, T4 DNA ligase (Promega Corp., Madison, USA) was added in each extract according to the manufacturer’s instructions. Outward amplification with PCR primers (Additional File 2) covering portions of the *bla*_OXA-72_ region was carried out under standard conditions. The product was then sent for sequencing at Mission Biotech, Taipei, Taiwan.

### Determination of the contribution of *bla*_OXA-72_ to carbapenem MIC

The *bla*_OXA-72_ was cloned into the shuttle vector pYMAb2 [21], which carries a kanamycin resistant determinant. The recombinant plasmid (pYMAb2:: *bla*_OXA-72_) and control plasmid (pYMAb2) were then transformed into the kanamycin-susceptible Ab290 strain by electroporation using an gene pulser electroporator (Bio-Rad, Hercules, CA) and 2 mm electrode gap cuvettes. The antimicrobial MICs of Ab290 (pYMAb2:: *bla*_OXA-72_) and Ab290 (pYMAb2) were determined as previously described [15]. Ab290 was an *A. baumannii* clinical isolate that was susceptible to multiple antimicrobial agents from Taipei Veterans General Hospital and has been used for transformation in the previous study [19]. After incubation without or with antimicrobial agents, the OXA-72 mRNAs in Ab290 (pYMAb2:: *bla*_OXA-72_) were compared using quantitative PCR [21]. Briefly, bacterial RNA was extracted using an RNAprotect Bacteria Reagent and RNeasy mini-kit (Qiagen, Valencia, CA). After elimination of genomic DNA by RNase-free DNase (Qiagen) treatment, reverse transcription was performed using random hexamers (Qiagen). Real-time PCR for *bla*_OXA-72_ was carried out with *recA* as an internal control on an ABI 7500 Fast real-time PCR system (Applied Biosystems, Inc.) These experiments were performed in at least triplicate.

## Results

### Bacterial identification and antimicrobial susceptibility testing

One-hundred and forty-two *A. baumannii* strains were resistant to imipenem. Among them, 27 (19.0%) carried *bla*_OXA-72_, including 22 of 38 isolates from Southern Taiwan (57.9%) and 5 of 62 isolates from Central Taiwan (8.1%). The number of isolates with *bla*_OXA-72_ was significantly higher in those obtained from the southern area than those from other areas (57.9% vs. 4.8%, respectively; *P* < 0.001 by χ2 test). All of the *A. baumannii* isolates carrying *bla*_OXA-72_ were resistant to carbapenems (Table 1). In addition, they were all multidrug resistant [22], but were susceptible to colistin.

**Table 1:** **Minimal inhibitory concentrations (MICs) of antimicrobial agents for *Acinetobacter baumannii *carrying *bla***_**OXA-72**_

**Antimicrobial agents**	**Isolates carrying *bla***_**OXA-72 **_**(*n*** **= 27)**		
	**MIC50 (mg/L)**	**MIC90 (mg/L)**	**Resistance rate (%)**
Imipenem	128	128	100.0%
Meropenem	>128	>128	100.0%
Ticarcillin	>128	>128	100.0%
Piperacillin	>128	>128	100.0%
Ceftazidime	>128	>128	100.0%
Cefepime	64	>128	96.3%
Sulbactam	32	32	63.0%
Ciprofloxacin	128	>128	100.0%
Colistin	1	1	0.0%

### Identifying Ambler class B metallo-β-lactamases and class D β-lactamases

All isolates with *bla*_OXA-72_ had the *bla*_OXA-51-like._ The *bla*_OXA-23-like_, *bla*_OXA-58-like_, *bla*_OXA-143,_*bla*_IMP-like_, *bla*_VIM-like_, *bla*_GIM-1_, *bla*_SPM-1_ and *bla*_SIM-1_ genes were not detected by PCR methods. In addition, IS*Aba1* was not detected in any of the strains.

### Determination of clonality by pulsed-field gel electrophoresis

**Figure 1:**
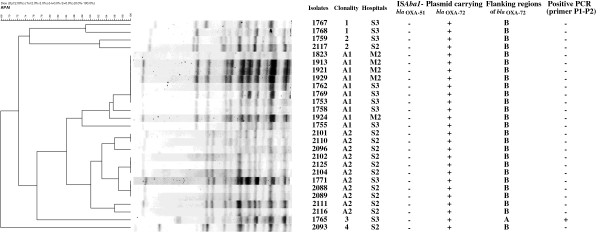
**Molecular characteristics of *Acinetobacter baumannii *carrying *bla***_**OXA-72 **_**in Taiwan.** The results of pulsed-field gel electrophoresis are shown, followed by the hospitals of the isolates, pulsotype, presence of IS*Aba1* upstream of *bla*_OXA-51_, localization of *bla*_OXA-72_, flanking regions of *bla*_OXA-72_ (delineated in Figure 3), and results of PCR using primers previously described.

To determine whether emergence of these isolates with *bla*_OXA-72_ was due to clonal spread, pulsed-field gel electrophoresis was performed (Figure 1). Twenty-one isolates (77.8% belonged to 2 major clones (A1 and A2) (Figure 1). The other six isolates carrying *bla*_OXA-72_ (including isolate 1765) did not belong to the two major clones and had only 65-75% homology related to the major clones. The A1 clone comprised all (5/5, 100%) of the isolates from Central Taiwan and 5 isolates (5/10, 50.0%) from S3 hospital, whereas the A2 clone comprised 10 isolates (10/12, 83.3%) from S2 and 1 isolate (1/10, 10%) from S3 hospital.

### Determination of the plasmid localization of *bla*_OXA-72_ by Southern blot

**Figure 2:**
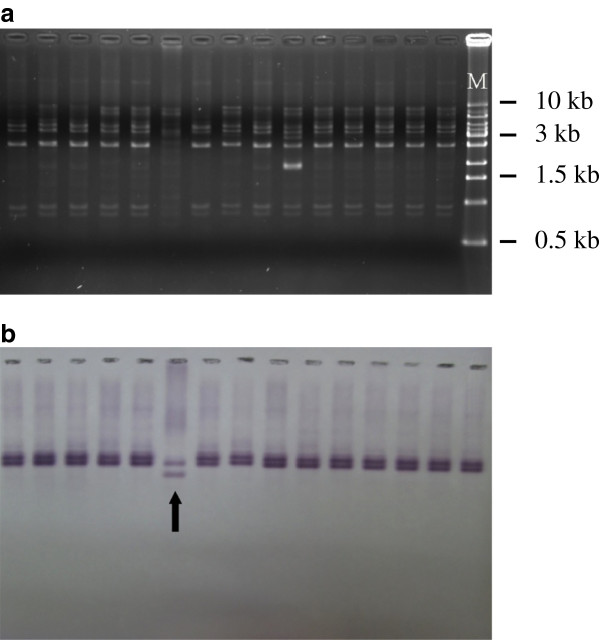
**Electrophoresis (a) and Southern blot (b) of isolates carrying *bla***_**OXA-72.**_ The pattern of plasmid electrophoresis and Southern blot was very similar among isolates. However, Southern blot revealed *bla*_OXA-72_ in isolate 1765 (arrow) may be located in a different genetic background. All isolates were tested but only the results of 15 isolates (from Lane 1 to 15) were presented. M, marker (Bio-1KB^TM^ Mass DNA Ladder, Protech Technology Enterprise Co., Ltd, Taiwan).

Electrophoresis showed minor differences in plasmid patterns among isolates (Figure 2). Southern blot using a *bla*_OXA-72_ probe further revealed the presence of *bla*_OXA-72_ in the plasmids obtained from 27 isolates. However, Southern blot also detected two types of segments flanking *bla*_OXA-72_ after *Eco*RI digestion. One was present in isolate 1765 and the other was present in the remaining 26 isolates.

### Sequencing of the flanking region of plasmid-borne *bla*_OXA-72_ by inverse PCR

**Figure 3:**
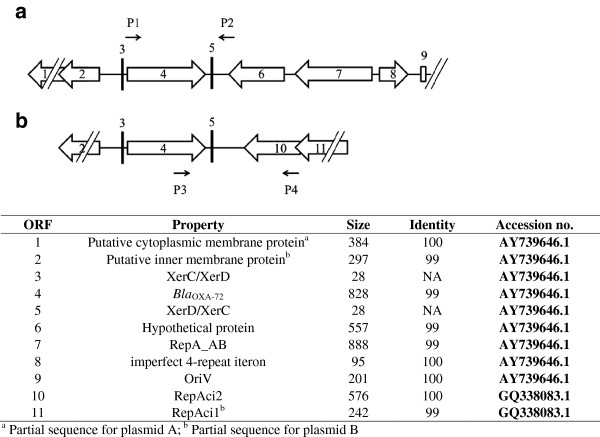
**Schematic map of the partial flanking region of *bla***_**OXA-72 **_**in two types of plasmids (plasmid A and B, Figures**3**a and**3**b, respectively) in Taiwan.** The arrows indicate the predicted open reading frames (ORFs) and their transcriptional directions. The names of various features are indicated below the map. The sequenced fragment in plasmid A shared 99% similarity to the previously prevalent plasmid (accession no. **AY739646.1**), whereas the region downstream of XerD/XerC in plasmid B was identical to another plasmid (accession no. **GQ338083.1**). P1 and P2 primers are specific for plasmid A and P3 and P4 primers are specific for plasmid B.

Based on the Southern blot results, we anticipated that there were at least two genetic structures flanking the *bla*_OXA-72_ gene. Inverse PCR of isolate 1765 and randomly selected isolates revealed two types of genetic structures flanking the gene (Figures 3a and 3b, fragment A of 3596 bp and B of 2325 bp). An identical 967 bp region containing *bla*_OXA-72_ in both fragment A and B was flanked by XerC/XerD and XerD/XerC. The 3596 bp fragment A had 99% similarity to pAB02 (GenBank accession no. **AY228470.1**) and contained a region with 100% similarity to the plasmids reported from the same teaching hospital (828 bp, accession no. **AY739646.1**) [13]. In fragment B, the 1195 bp region upstream of XerD/XerC had 99% similarity to that in fragment A, but the region downstream of XerD/XerC was similar to other plasmids without *bla*_OXA-72_ (accession no. **GQ338083.1**), indicating possible recombination between the plasmids. Using a specific primer pair (P3 and P4, Additional File 2) for fragment B (Figure 3), the PCR result was positive in 26 isolates, except isolate 1765. The specific primers used to confirm the presence of fragment A were similar to those used in a previous study (primers P1 and P2, respectively; Figure 3) [13]. Only one isolate (isolate 1765) was positive for fragment A. The mutual exclusion of fragment A and B indicated that they were located on different plasmids, designated as plasmid A and B, respectively.

### Determination of the contribution of *bla*_OXA-72_ to carbapenem MIC

**Figure 4:**
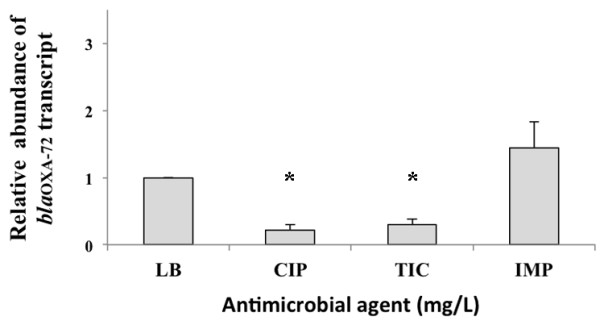
**Results of quantitative PCR of mRNA levels of OXA-72 in *Acinetobacter baumannii *carrying *bla***_**OXA-72 **_**in response to different antibiotics.** The *A. baumannii* isolate carrying *bla*_OXA-72_ was treated without antibiotics (Luria-Bertani broth, LB), or with ciprofloxacin (CIP) at 0.25 mg/L, ticarcillin (TIC) at 1024 mg/L, and imipenem (IMP) at 8 mg/L for 7 hours. Quantitative PCR revealed higher mRNA levels of OXA-72 in isolates treated with imipenem compared to those treated with ciprofloxacin or ticarcillin. * *P* value < 0.05 by one-way analysis of variance (ANOVA) and by the Scheffe post-hoc method.

To determine the contribution of *bla*_OXA-72_, *A. baumannii* strain Ab290 was transformed with pYMAb2 and pYMAb2::*bla*_OXA-72_, respectively. Compared to Ab290 (pYMAb2), transformation with *bla*_OXA-72_ resulted in an increase in the MICs of imipenem, meropenem, ticarcillin, sulbactam, and ampicillin (Table 2). We were interested in determining if the addition of carbapenems would increase OXA-72 expression. Thus, strain Ab290 (pYMAb2::*bla*_OXA-72_) was treated with ciprofloxacin, imipenem, and ticarcillin. Quantitative PCR showed that the RNA expression of the gene increased by 8-fold after addition of imipenem compared to addition of ciprofloxacin or ticarcillin (Figure 4).

**Table 2:** **Minimal inhibitory concentrations (mg/L) of antimicrobial agents for *Acinetobacter baumannii *with or without plasmids carrying *bla***_**OXA-72**_

**Isolates**	**Imipenem**	**Meropenem**	**Ticarcillin**	**Ampicillin**	**Sulbactam**	**Cefepime**	**Ceftazidime**	**Ciprofloxacin**
Ab290	0.25	0.5	8	128	2	2	2	0.5
Ab290 (pAbYM2)	0.25	0.5	8	128	4	2	4	0.25
Ab290 (pAbYM2::*bla*_OXA-72_ )	32	32	>1024	>1024	16	4	4	0.5

## Discussion

The resistance rate to carbapenems in *A. baumannii* has been increasing worldwide. Ambler class D β-lactamases are responsible for the main mechanism of resistance in *A. baumannii*. *Bla*_OXA-72_, which was first discovered in Thailand, has since spread rapidly to other areas of Asia and Europe [9-13]. In this study we collected 142 IRAB from different geographical regions of Taiwan. Among them, 27 IRAB carrying *bla*_OXA-72_ gene had successfully spread throughout Southern Taiwan and extended into Central Taiwan*.* One new type of plasmid, which harbored a different genetic structure compared to that previously reported, not only existed in the two major clones, but also disseminated to other clones. We also found evidence that *bla*_OXA-72_ contributed to carbapenem resistance. The mRNA levels of OXA-72 increased in response to the addition of imipenem.

Many studies have demonstrated an association of carbapenem resistance and the presence of *bla*_OXA-72_. One study [23] revealed that the plasmid carrying *bla*_OXA-72_ modestly increased the MICs of imipenem and meropenem in *Escherichia coli* (0.125 to 0.25 and 0.016 to 0.032, respectively). Another study [9] showed that transformation of susceptible strains with the entire plasmid carrying *bla*_OXA-72_ extracted from clinical isolates resulted in resistance to carbapenems, but not cephalosporins. However, the increase of carbapenem MIC might be attributed to other genetic determinants also present in the transformed plasmid in that study. In our study, we only cloned the *bla*_OXA-72_ gene into the plasmid without other resistance mechanisms against carbapenems. The transformation greatly increased the MICs of carbapenems (64-128–fold), which was further supported by the elevated mRNA levels of OXA-72 in response to the addition of imipenem. Moreover, our study has revealed for the first time that the transformation also increased the MICs of ticarcillin, ampicillin, and sulbactam.

The molecular epidemiology of Ambler class D β-lactamases differs among geographical regions [7]. Our previous study showed prevalence of *bla*_OXA-23_ in Central Taiwan [24], whereas the current study showed the *bla*_OXA-72_ was mainly disseminated in Southern Taiwan. Our study revealed that the clonal spread and plasmid dissemination were important for *bla*_OXA-72_, similar to previous studies [13,23,25]. The mechanisms of spread of different carbapenemase genes seemed to be different, as our previous study showed that a transposon may be the main reason of spread of *bla*_OXA-23_ in Central Taiwan [24].

Lu *et al.* found that the plasmid carrying *bla*_OXA-72_ was positive for PCR in half of the isolates (49.2%, 29/59) in one teaching hospital in Southern Taiwan using P1 and P2 primers [13]. Our study recovering isolates from multiple centers only identified one isolate from the same hospital assessed in a previous study [13] that may harbor the same plasmid (plasmid A). This suggests that plasmid A may have been replaced by plasmid B during this period. Plasmid B shared part of the genetic structure with plasmid A. The shared regions included not only regions between XerC/XerD binding sites, which were presumed to be responsible for mobilization of *bla*_OXA-72_[23,25,26], but also regions upstream from it. This may suggest that these plasmids have undergone a recombination event.

## Conclusions

In conclusion, our study has revealed that the dissemination of IRAB isolates carrying the *bla*_OXA-72_ gene may be due to clonal spread and dissemination of a new plasmid. The *bla*_OXA-72_ gene contributed to carbapenem resistance.

## Abbreviations

CLSI: Clinical and laboratory standards institute; MIC: Minimal inhibitory concentration; IRAB: Imipenem-resistant *Acinetobacter baumannii*.

## Competing interests

The authors declare that they have no competing interests.

## Authors’ contributions

SCK and SPY conceived of the study, and participated in its design and coordination and drafted the manuscript. YTL and HCC participated in the sequence alignment, and drafted the manuscript. CPC and CLC carried out the laboratory assays. PLL and PRH collected the clinical isolates. TLC and CPF designed and supervised the study, participated in data analysis and interpretation, and finalized the manuscript. All authors read and approved the final version of the manuscript.

## Pre-publication history

The pre-publication history for this paper can be accessed here:

http://www.biomedcentral.com/1471-2334/13/319/prepub

## Supplementary Material

Additional file 1 **Distribution of *Acinetobacter baumannii *carrying *bla *OXA-72 among 291 *A. baumannii *isolates collected from 10 teaching hospitals in Taiwan (N, northern; C, central; S southern).** Click here for file

Additional file 2 Primers used in this study. Click here for file
