# Supplementary material for: Dissemination of imipenem-resistant Acinetobacter baumannii with new plasmid-borne blaOXA-72 in Taiwan
Source: BMC Infect Dis. 2013 Jul 13;13:319. doi: 10.1186/1471-2334-13-319 (PMC3728158; doi:10.1186/1471-2334-13-319)
Supplement: Additional file 1 — Distribution of Acinetobacter baumannii carrying bla OXA-72 among 291 A. baumannii isolates collected from 10 teaching hospitals in Taiwan (N, northern; C, central; S southern). [file 1471-2334-13-319-S1.pdf]

Supplementary Figure S1

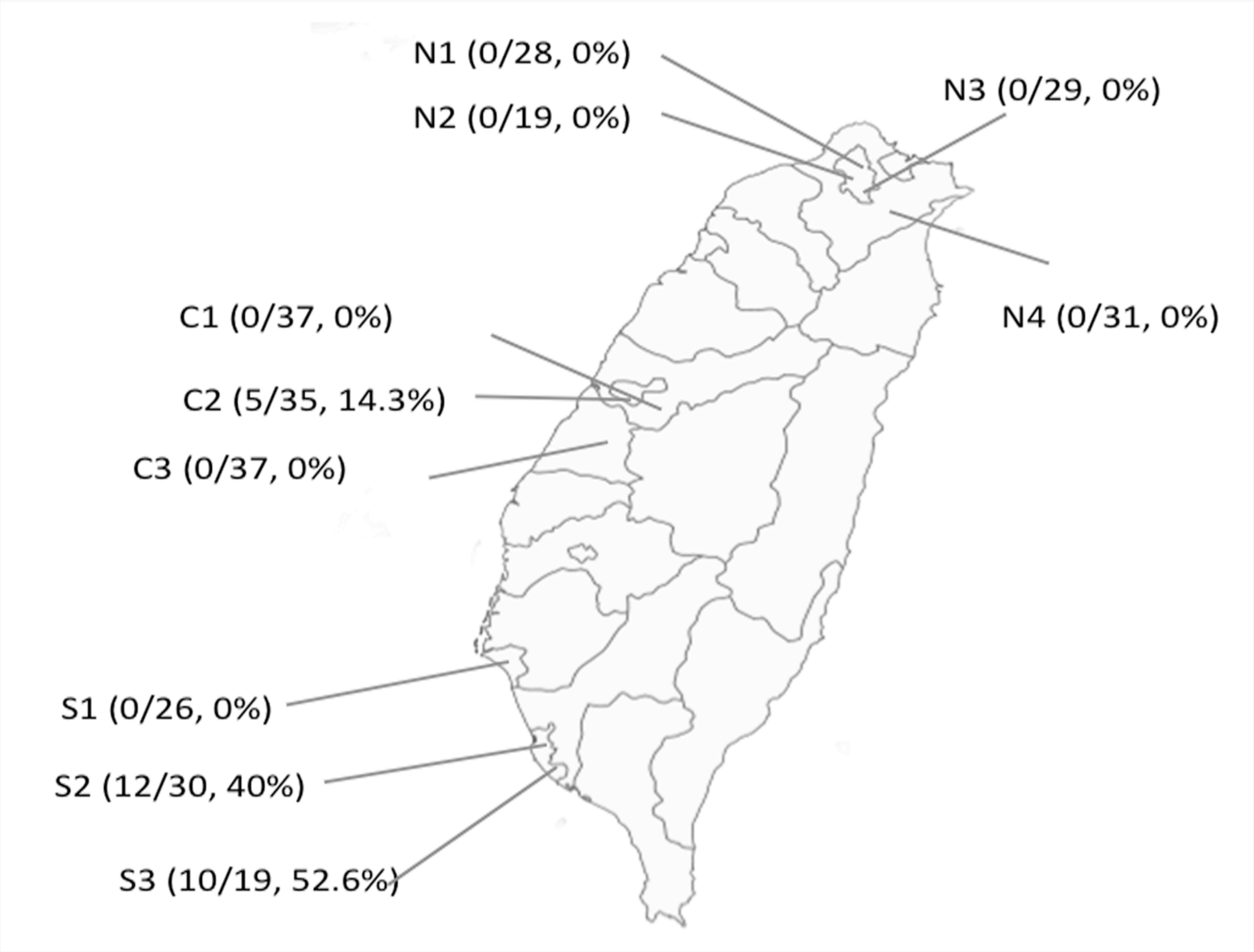

Supplementary figure legend

Figure S1. Distribution of *Acinetobacter baumannii* carrying $bla_{OXA\text{-}72}$ among 291 *A. baumannii* isolates collected from 10 teaching hospitals in Taiwan (N, northern; C, central; S southern).
