# Supplementary material for: Dissemination of imipenem-resistant Acinetobacter baumannii with new plasmid-borne blaOXA-72 in Taiwan
Source: BMC Infect Dis. 2013 Jul 13;13:319. doi: 10.1186/1471-2334-13-319 (PMC3728158; doi:10.1186/1471-2334-13-319)
Supplement: Additional file 2 — Primers used in this study. [file 1471-2334-13-319-S2.doc]

Supplementary Table S1. Primers used in this study,

| **Targets** | **Primer names** | **Sequences (5’-3’)** | **References** |
| --- | --- | --- | --- |
| *bla_OXA-23-like_* | m23F | GATCGGATTGGAGAACCAGA | 6 |
|  | m23R | ATTTCTGACCGCATTTCCAT | 6 |
| *bla_OXA-24-like_* | m24F | GGTTAGTTGGCCCCCTTAAA | 6 |
|  | m24R | AGTTGAGCGAAAAGGGGATT | 6 |
| *bla_OXA-51-like_* | m51F | TAATGCTTTGATCGGCCTTG | 6 |
|  | m51R | TGGATTGCACTTCATCTTGG | 6 |
| *bla_OXA-58-like_* | m58F | AAGTATTGGGGCTTGTGCTG | 6 |
|  | m58R | CCCCTCTGCGCTCTACATAC | 6 |
| *bla_IMP_* | Imp-F | GGAATAGAGTGGCTTAAYTCTC | 17 |
|  | Imp-R | CCAAACYACTASGTTATCT | 17 |
| *bla_VIM_* | Vim-F | GATGGTGTTTGGTCGCATA | 17 |
|  | Vim-R | CGAATGCGCAGCACCAG | 17 |
| *bla_GIM-1_* | Gim-F | TCGACACACCTTGGTCTGAA | 17 |
|  | Gim-R | AACTTCCAACTTTGCCATGC | 17 |
| *bla_SPM-1_* | Spm-F | AAAATCTGGGTACGCAAA | 17 |
|  | Spm-R | ACATTATCCGCTGGAACAGG | 17 |
| *bla_SIM-1_* | Sim-F | TACAAGGGATTCGGCATCG | 17 |
|  | Sim-R | TAATGGCCTGTTCCCATGTG | 17 |
| IS*Aba1* | ISAba1F | CACGAATGCAGAAGTTG | 6 |
|  | ISAba1R | CGACGAATACTATGACAC | 6 |
| *bla*_OXA-72_ for Southern blot | SB72F | CTTCCTATATTCAGCATTTC | this study |
|  | SB72R | CCAAGATTTTCTAGCGAC | this study |
| Inverse PCR for *bla*_OXA-72_ | INV72F | ACCCTGTGTTTGAGCTTCATC | this study |
|  | INV72R | GCCTTATGCGAAATGCTTGA | this study |
| Primers specific for plasmid A | P1 | TTCCCCTAACATGAATTTG | 13 |
|  | P2 | GTACTAATCAAAGTTGTGAA | 13 |
| Primers specific for plasmid B | P3 | TTATGCAAAAAGTGGATGGGGA | this study |
|  | P4 | GCTCCAGTGATGGACAATTCGA | this study |
